# Perceived Stigma as a Contextual Barrier to Early Uptake of HIV Testing, Treatment Initiation, and Disclosure; the Case of Patients Admitted with AIDS-Related Illness in a Rural Hospital in South Africa

**DOI:** 10.3390/healthcare9080962

**Published:** 2021-07-29

**Authors:** Sphiwe Madiba, Evelyn Ralebona, Mygirl Lowane

**Affiliations:** 1 Department of Environmental and Occupational Health, School of Public Health, Sefako Makgatho Health Sciences University, Pretoria 0001, South Africa; ralebonae@gmail.com; 2 Department of Biostatistics, School of Public Health, Sefako Makgatho Health Sciences University, Pretoria 0001, South Africa; mygirl.lowane@smu.ac.za

**Keywords:** perceived-stigma, HIV/AIDS, disclosure, family, HIV testing, concealment, rural, South Africa

## Abstract

We explored the extent to which perceived HIV-related stigma influences the disclosure and concealment of HIV status to family among adult patients hospitalised for AIDS-related illness, and described reports of negative responses and enacted stigma following disclosure. We conducted interviews with a purposeful sample of 28 adult patients in a rural South African hospital. Data analysis was deductive and inductive and followed the thematic approach. We found evidence of delayed HIV diagnosis and initiation of treatment. There was delayed and selective disclosure as well as concealment of the HIV-positive status. The disclosure was delayed for months or even years. During that time, there was active concealment of the HIV status to avoid stigma from family, friends, and community. When disclosure occurred, there was selective disclosure to close family members who would keep the secret and respond favorably. Although the participants disclosed mostly to close family, some of their post-disclosure experiences included incidents of enacted stigma and discrimination. The fear of perceived stigma and self-stigma influenced the active concealment of their HIV status from others. Continuous concealment of one’s HIV status and delayed disclosure limit the opportunities for support and care. There is a need to take into consideration the interaction between HIV-related stigma and disclosure to develop disclosure-counselling strategies in primary health care settings.

## 1. Introduction

Disclosure is a very challenging step that people have to take after being diagnosed with HIV. In both low-and high-resource settings, many people with a positive HIV diagnosis face difficulties regarding when and how to reveal their status to those around them [1]. People living with HIV (PLHIV) also struggle with how to cope with the possible negative consequences of disclosing [2]. Disclosure creates uncertainty about potential responses, since the individual who discloses cannot predict the response of the recipients [3,4]. PLHIV risk receiving a negative response if they disclose, which may be due to the stigma that surrounds HIV [2,5].

To minimise the potential risks of disclosure, some PLHIV conceal or do not disclose their HIV-positive status. Since disclosure is a process, in most instances disclosure does not occur instantaneously but occurs over time [6]. Therefore, when PLHIV select people to disclose to, they weigh the people’s potential reactions to the disclosure and anticipate the effects of the disclosure on others [7]. As such, when disclosure to family and friends occurs, it is a deliberate, selective, and often planned behaviour, which balances risks and benefits [8,9,10]. The disclosure of one’s HIV-positive status has important public health implications [2]. For individuals, disclosure to family and friends provides a way of obtaining social and psychological support, decreasing anxiety, improving psychological well-being, increasing access to clinical care, early enrollment on antiretroviral therapy ART), and better adherence to the therapeutic regimen [1,9,11,12,13].

PLHIV disclose to family or friends in anticipation of the supportive responses they might receive [11,14]. However, the responses from the people they disclose to may include both support and rejection [15,16]. Consequently, disclosure is withheld or concealed because of the fear of rejection, stigma, discrimination, and abandonment by family, friends and sexual partners [11,17]. The literature suggests that the fear of stigma and the forms of discrimination associated with it contribute to non-disclosure in many settings [2,18,19,20]. Perceived stigma concerns the subjective awareness of stigma in society or the community. When PLHIV experience a high degree of perceived stigma, they are more likely to protect themselves by avoiding disclosure [21].

PLHIV and those close to them are affected by all the forms of stigma; self-stigma, perceived stigma, and enacted/experienced stigma and discrimination [21], particularly in Southern Africa, where the burden of the HIV epidemic is significant [22]. HIV stigma evokes significant fear in others, and the impact of stigma on the uptake of HIV testing has been reported in several studies [23,24]. Perceived stigma and the fear of receiving a positive test result are the factors most often cited as influencing the uptake of HIV testing [25,26,27]. Late HIV diagnosis and presentation to care increases the risk of increased HIV transmission, poor health outcomes, and poor response to ART, increased hospitalisation, and increased health care costs [26,28,29].

Research regarding disclosure to family and friends in sub-Saharan Africa is limited and there is evidence that the rate of disclosure to family in poorly resourced settings, including South Africa, remains low [11,14,30]. It should be noted that disclosure can result in social support for PLHIV when family and friends react positively to the disclosure. Although PLHIV may disclose to family members because of their close relationship as well as the social support they expect to gain [31], Evangeli and Wroe [13] have noted that clinical and empirical findings suggest that family can be a significant source of stress for PLHIV. Gilbert and Walker [23] assert that fear of stigma and rejection is the main reason why PLHIV do not want to disclose their status, especially to family members.

Despite the deterrents and barriers against disclosure to family, the social support function of HIV status disclosure to family is vital for PLHIV [24]. While disclosure is crucial when AIDS-related illnesses set in and the risk of infecting family members who often play the role of caregivers is high, the focus of disclosure studies is on healthy adults, particularly women [14,15]. Few studies explore the experiences and outcomes of the disclosure of one’s HIV status to family [11], particularly in settings that bear a disproportionate burden of the HIV epidemic, like South Africa [24]. Even fewer studies investigate disclosure to family in rural settings, and even less is known about the reasons for nondisclosure to other social groups such as children and friends [14].

There is insufficient understanding of the influence of stigma in the adoption of HIV care and HIV disclosure [32]. Research shows that over one-third of the HIV infected individuals in sub-Saharan Africa present to HIV/AIDS care late and encounter many problems, such as poor treatment outcomes, increased mortality, high healthcare costs, and the development of opportunistic infections [33]. Furthermore, delayed disclosure, non-disclosure, and concealment could lead to psychological stress that could interfere with the medical care PLHIV need [34].

In this study, we explore the extent to which perceived HIV-related stigma influences disclosure and the concealment of HIV status to family members and sexual partners among adult patients hospitalised for AIDS-related illness. The secondary objective is to examine and describe patients’ reports of negative responses and acts of stigma following disclosure of their HIV status. Given the emphasis on HIV testing and treatment in Africa, there is a need to understand how PHIV react to their diagnosis and reach out for support to significant others in their network [24].

## 2. Materials and Methods

### 2.1. The Design and Setting of the Study

The study used an explorative qualitative design involving the performance of individual in-depth interviews with patients admitted in medical wards in a rural hospital. The hospital where the study was conducted is about 130 km from Polokwane, the capital of Limpopo Province. The hospital serves a population of about 283,307 for all health- related conditions. Amongst other wards, there are two main medical wards, one female and one male, which receive approximately 30 patients monthly for HIV/AIDS-related illnesses.

The study sample consisted of adult patients admitted to the two wards with AIDS-defining illnesses. The lead author used purposive sampling to select patients for one-on-one interviews. Those who met the inclusion criteria were identified with the assistant of the ward managers and nursing staff. The patients were selected if they were 18 years and above, not terminally ill, and able to provide informed consent. An attempt was also made to achieve variety in the sample in terms of age, gender, marital status, employment status, time since diagnosis, and duration on ART. The size of the sample was influenced by data saturation, which was achieved after 28 interviews had been conducted. Data is considered to have been saturated when subsequent interviews no longer generate new information to contribute to the understanding of the phenomenon under investigation [35,36].

### 2.2. Data Collection

The main author and a research assistant conducted one-on-one in-depth interviews with the participants. The research assistant was trained on the objectives of the study, on the sensitive nature of the topic, and on the interview guide by the second author (SM). An interview schedule with open-ended questions was used. The development of the tool had been informed by previous research on disclosure. It was developed in English and translated into Sepedi, the local language spoken by the population in the setting of the study. The interview guide included questions relating to HIV testing and disclosure practices, the participants’ response to testing positive, disclosing to their families, what motivated them to disclose, their families’ responses to the disclosure, or what deterred them from disclosing. In addition, probing elicited further data, and follow-up questions were asked to extend the understanding of the issues at play.

All the interviews were conducted after the patients had been seen by the doctor and ward rounds had been completed, in order to avoid the disruption of the ward routine. The interviews took place in a room provided by the unit manager to ensure privacy and confidentiality. Informed consent was obtained from all the participants before the interviews began, and they were informed about the voluntary nature of their participation and their right to withdraw from the study at any stage. Each interview lasted for 40 to 50 min, and was recorded after permission to record had been obtained from the participants. Using the local language and engaging in probes allowed the participants to provide detailed and rich responses about their experiences of disclosure. Brief demographic and clinical data were collected from each participant after each interview. The data were used to describe the cohort of participants in such a way as to give context to the qualitative data.

### 2.3. Data Analysis

The audio-recorded data from the in-depth interviews were transcribed verbatim in Sepedi and translated into English by the lead author and the research assistant. The lead author checked the translated transcripts for accuracy against the audio recordings. The data were analysed using thematic analysis following the inductive and deductive approach as outlined in Braun and Clarke [37]. The authors independently read a few transcripts repeatedly to familiarise themselves with the data and identify emerging codes. The authors then used the initial codes and emerging themes to develop a codebook, meeting several times to agree on the definition of themes and subthemes. After they had reached consensus, the transcripts were then imported into Nvivo11, the qualitative analysis software package that was used for the data analysis [38]. Analysis continued until the themes and sub-themes that described the disclosure experiences of the patients had been identified.

To ensure rigour, several strategies pertaining to credibility, dependability, transferability, and confirmability were used [39]. We conducted the interviews in the language of the participants, used a good digital recorder, and transcribed verbatim. The lead author kept an audit trail throughout the research process, and used NVivo computer software for data analysis. Lastly, all the authors were involved in the data analysis to minimise interpretation bias.

### 2.4. Ethical Considerations

Sefako Makgatho Health Sciences University Research Ethics Committee (SMUREC) granted approval for this study (SMUREC/H/158/2016: PG). Permission to conduct the study was granted by the hospital management. All the participants were informed that their participation was voluntary and about the confidentiality of the data. Informed concern was obtained before beginning the interviews.

## 3. Results

The sample consisted of 28 patients hospitalised for AIDS-related opportunistic illnesses, most of whom (15/28) had repeated admissions. Their ages ranged from 20 to 60 years (median, 45 years), 15/28 were 40 years and above, 18/28 were females, 15/28 were single, 21/28 were unemployed, 18/28 had children, only three of them lived alone, and the source of income for most (17/28) was the social grant. With respect to HIV-related data, 20/28 had tested for HIV while they were experiencing the symptoms of HIV/AIDS. Most, (13/28) had known about their HIV diagnosis for more than 5 years (range 1–16 years), 8/28 had known their HIV diagnosis for one year, and yet they had been hospitalised for opportunistic diseases, indicating late uptake of HIV testing. With respect to receiving ART, most (13/28) had been on lifelong ART for less than a year, with only 3/28 reporting to having received ART for 6–10 years. This suggests late presentation in a health facility to initiate HIV care, but also poor levels of adherence, as 10/23 were not adherent. This section may be divided by subheadings. It should provide a concise and precise description of the experimental results, their interpretation, as well as the experimental conclusions that can be drawn (Table 1).

### 3.1. Themes

Six main themes emerged from the analysis of the interviews namely: delayed testing for HIV, selective disclosure of the HIV status, endorsing the secret nature of the HIV diagnosis, delayed disclosure, disclosure to receive appropriate care, and negative responses to the disclosure. Four sub-themes emerged under the theme delayed disclosure (Table 2).

#### 3.1.1. Delayed Testing for HIV

Fear of the possible consequences of the HIV diagnosis was an important reason for late diagnosis. The participants indicated that they had undertaken an HIV test after they experienced severe, persistent, and often unexplained illness. Most of them tested when they or their partners were sick, and a few tested after the death of their partners. For some, the knowledge of a partner’s HIV status was a driving force for testing.

I tested because my boyfriend with whom I was cohabiting died after he was sick for long. I did not know what his problem was, but my family told me to go to the clinic and test.

(31-year-old female)

After my husband passed away, I suspected that he had AIDS. I then decided to test.

(57-year-old female)

I used to take my wife to the doctor because she was always sick. The doctor said that he was worried because we came repeatedly for consultation and he suggested that my wife be tested. When she tested positive, the doctor insisted that I also test at the same time and I tested HIV-positive.

(48-year-old male)

I came to the hospital because I had chest pains. The pain was so much that I could not do anything… That is when I said, let me test.

(34-year-old female)

#### 3.1.2. Selective Disclosure of HIV Status

Those who indicated that they had disclosed selected family members they were close to and whom they perceived would offer them support. These included parents, siblings, children, and other close family members such as aunts and sisters-in-law. The persons most frequently disclosed to were family members with whom they lived in the same household.

I told my younger sister, I told her that I am sick, that I was suffering from this disease (HIV). My younger sister said I must tell our parents at home and I said I would tell them when I feel free. Then I told my brother, who told my mother.

(37-year-old female)

I told my sister and my brother. My sister is the person who sometimes prepares food for me to eat. Therefore, I cannot keep a secret in her home, and my brother helps me with cash when I do not have enough.

(45-year-old male)

I told my family after my boyfriend passed away. I went home specially and called the family. I told them that I am living with HIV.

(33-year-old female)

As indicated, the participants selected with whom to share their status and concealed their status from some family members, whom they perceived could harm them.

I did not tell other family members… Some of the family members do not treat you well when you are sick. Those who look down at us should not be told.

(45-year-old male)

I will not tell them… Even my mother said that we should not tell the family. You know… some families fight, you will find them pointing fingers at you, and you find yourself no longer enjoying… You have stress and start defaulting your treatment.

(34-year-old female)

I cannot tell my in-laws because they are the first ones who will tell people that I had killed their brother… They won’t say I contracted it (HIV) from their brother, but will say I killed their brother, so I just leave it like that.

(57-year-old female)

#### 3.1.3. Endorsing the Secret Nature of the HIV Diagnosis

The selection of family members to disclose to meant that the participants trusted them to keep the secret of the HIV diagnosis.

I disclosed after being sick… I understood that my mother would not go around telling people.

(36-year-old female)

HIV is not something that has to be disclosed to everyone, it is a secret. The disease is only known by my family. It will be wrong if many people can know about this disease.

(60-year-old male)

We only have to know about the disease (HIV) as a family… People at home should not disclose to strangers, only the family should know. You cannot tell people all your secrets.

(36-year-old-female)

#### 3.1.4. Delayed Disclosure

Of the 28 participants, only five had not disclosed their HIV status to anyone, while 23 had disclosed, albeit disclosure for most was delayed. The time span between the HIV test and the disclosure of the HIV status varied from days to months and even to years. They stated that disclosure to family members occurred sometime after the diagnosis of HIV. The barriers to early disclosure most frequently referred to included wanting to protect their dignity, the fear of stigma, and the belief that an opportunity to disclose would present itself.

##### Perceived Stigma

The participants’ fears of stigma and the consequences of having a positive HIV status resulted in delayed disclosure to close family members. The fear and expectation of discrimination led to delayed and selective disclosure or to opting not to disclose at all.

I won’t tell because you will find your issues all over the place. They will go around telling people in the village that he is HIV-positive.

(52-year-old male)

It is just to preserve your dignity. When people know that I have HIV they will say it means that the time when she was in university she was sleeping around with men. That is why she is HIV-positive. It is about dignity.

(45-year-old female)

My family do not know anything. We do not talk to each other. When I left home, we were not in good terms. They will just go around gossiping.

(34-year-old female)

I do not think it is good to tell people about your HIV status while it is known that many people do not like the disease.

(45-year-old male)

It is because they (people) talk badly and you just understand that now you will live being isolated from other people.

(45-year-old female)

##### Self-Stigma

Some of the older participants viewed their HIV status as being shameful, because of their age (15/28 were 50 years and older), which suggests that they had internalised the HIV and accepted the negative views that others in society might hold about them. Social stigma, whether real or perceived, had impeded disclosure among the participants.

This disease (HIV) made me to be ashamed of myself.

(45-year-old male)

Hey, this issue doesn’t sit well with me… Eish! In fact, this issue is a difficult issue. I, it is just that, it makes me to be ashamed. AIDS is a disease that is shameful.

(47-years-old male)

It is shameful… To tell the truth I was shameful, the shame of this thing (HIV), if you know how this thing (HIV) came. Now you have to tell the children something like this. They talk about HIV- positive people in a bad way.

(57-year-old female)

##### Disclosure Delayed until When Symptoms Were Visible

Some of the patients, who had not disclosed to anyone in their families for a long time, did so when they became sick and felt compelled to tell their families. For some, disclosure was motivated by the desire to protect those who provided them with care and support. Some disclosed in response to relentless questions about their illness.

I told them, when I became sick like this. They sometimes bath me, and I did not want them to bath me without wearing anything on their hands. They must wear gloves.

(54-year-old female)

I disclosed when I became sick because they wanted to know what my problem was. It is then that I explained that I have something like this (HIV).

(37-year-old female)

I was sick all the time… I was not a sickly person, and they wanted to know the disease that I was suffering from. I told them that I am HIV-positive.

(34-year-old female)

##### Disclosure Delayed until after Admission in Hospital

Of the 28 participants, 13 disclosed after their hospitalisation, while some disclosed to their families whilst in the hospital. Their narratives indicated that they had no choice, but were forced to disclose to their families. They said they wanted to avoid them hearing about their HIV status from the health professionals in the wards.

My aunt called late yesterday after I was admitted. She wanted to know how I was. I told her that my situation is like this, when they (nurses) checked me, they told me that I have this disease (HIV).

(45-year-old female)

I told her (my wife) because I got admitted. I told her to come to the hospital, and when she got here, I showed her the (HIV positive) results.

(51-year-old male)

Normally I cannot say that come here let me tell you that I am suffering from this (HIV). If it is the issue of them being here (visiting the hospital) and worried that this man always gets sick then I have to tell them that I have this disease (HIV).

(48-year-old male)

#### 3.1.5. Disclosure to Receive Appropriate Care

The data further revealed that the participants disclosed to family members to receive support when needed it.

I told my children that the pills that I am taking are ARVs for HIV. I said, “You must know that your mother has this disease (HIV) but do not tell anyone.” When I am sick, you must tell the home-based caregivers that I am taking medication for HIV.

(45-year-old female)

All of my relatives know, especially because sometimes when there is a problem you have to call and tell them that you are not well, so that you get support.

(48-year-old male)

After I tested positive, they gave me many tablets. When I arrived at home, I called my children and showed them the tablets, and explained that they are for HIV. I told them that my life depends on them, and that they should not allow me to skip taking them.

(57-year-old female)

#### 3.1.6. Negative Responses to Disclosure

Perceived negative responses to disclosure have been identified as a deterrent to disclosure to families, partners, and other people in the network of HIV-positive individuals. A few participants narrated incidents of differential treatments, negative reactions, and being discriminated against by their families after disclosing.

After I told my sister-in-law about my disease (HIV), she would not share a couch with me anymore or talk with me the way I am sitting with you now. She thought I will infect her while talking to her. Although we live in the same yard, we do not ask for help from each other. The only thing that she said to me was that I must always have a phone with me because that phone will help me, as she does not care. She turned her back on me.

(37-year-old female)

They (the family) did not care. My mother said she does not want to see a corpse in her home. There was no one taking care of me. My brother took care of me when they said everyone must cook her own pot. She (her mother) wanted me to leave, so I left. I am staying with other people.

(34-year-old female)

I disclosed to all the family. I am sure I told them after three months. It is only now that they started treating me in a bad way since I started being sick. My sister is the one staying with me at home. When I tell her that I am feeling weak and ask her to call the ambulance for me, she does not care.

(33-year-old female)

My siblings, they are always acting weird. They say I will infect their children if I touch them.

(34-year-old female)

The five participants who disclosed to their partners described negative reaction from their partners upon disclosure.

I said let me start by telling this man that I am sleeping with (her sexual partner), but after I told him he left. He said that he is not HIV positive. He said it means I came with my disease. He left me and got another girlfriend and they stayed together.

(45-year-old female)

I cannot even tell you what we are fighting for. He just had some issues (her husband). As I said since this issue (HIV), he often insults me about it (her HIV status).

(47-year-old female)

We fought a lot with my wife. She was angry that I was hiding something so big (HIV), but now we do not have a problem.

(60-year-old male)

## 4. Discussion

This study explored HIV status disclosure and perceived negative responses to disclosure among adult patients hospitalised with AIDS-related illnesses. Opportunistic infections related to AIDS are the leading cause of hospitalisation for PLHIV, particularly in settings where HIV testing is delayed [40]. In the current study, 13 out of the 28 participants had been hospitalised several times with AIDS-related opportunistic diseases. Although the study did not investigate the reasons for the late diagnosis of HIV, the data showed that most of the participants tested for health-related reasons or because of the severity of their symptoms. Delayed HIV diagnosis has been reported in other studies that indicated that the symptoms of chronic illness compelled their participants to take HIV tests [31,40]. People present late for HIV testing because of a poor uptake of HIV testing services, fear of a positive result, and a fear of stigma [26,33,41,42]. Among older persons, HIV testing is delayed due to low self-perceptions of the risk of HIV transmission and acquisition [43].

We found widespread delayed and selective disclosure as well as concealment of HIV-positive status. The time it took the participants to disclose to their families is of great concern, given the potential individual and public health benefits associated with HIV disclosure [41]. Although research suggests that disclosure generally becomes easier the longer someone has been living with HIV and as she/he becomes more comfortable with their status [11], we did not find that. Some time elapsed before any disclosure for some of the participants. Prior research has reported similar observations [31]. The time span between HIV testing and disclosure varied from days to months and even to years. This was common among those who tested when they did not present with any symptoms. Consistent with previous research [7,24,44,45], some of the participants disclosed by necessity or were forced to disclose. Most (13/23) participants who had disclosed did so after they had been hospitalised or whilst in the hospital. The literature suggests that PLHIV who disclose at a more advanced stage of illness do so to get sympathetic support [46].

The motivations for disclosure in the current study are consistent with those found in previous studies [14,24]. Selective disclosure was determined by the nature and quality of the relationships as well as the social support expected [31]. Generally, PLHIV disclose to family members to receive social support. As already stated, most of the disclosure to family occurred long after the participants had tested HIV-positive and their medical condition had deteriorated. Therefore, at the time when they disclosed, there was a need for assistance with physical care. This study, like others [14,24], established the critical role that the family plays in providing support and care to adults infected with HIV/AIDS. Therefore, there is a need for a greater focus on disclosure to families for social support in HIV counselling intervention.

Research has noted that, while around two-thirds of PLHIV disclose, those who disclose do so selectively [1]. Selective disclosure is deliberate and involves weighing up the risks and benefits when choosing to disclose [10,24]. The participants in this study did not disclose to family members who would pose the highest risk of negative emotional consequences. As a disclosure approach, selective disclosure is used, particularly by older people, to protect their status and guard against stigma [43]. We observed more disclosure to siblings and parents than to sexual partners. Almost none of the participants had shared their HIV-positive diagnosis with anyone outside of the family. They emphasised that it was much safer to disclose to family members, whom they trusted to keep their HIV-positive status secret and thus to avoid stigma and discrimination. Prior studies have reported similar observations that PLHIV prefer to disclose to close family members before their sexual partners [7,14,20,24,34,47].

Steenberg [48] points out that PLHIV delay disclosure because of the fear of how their families might react to the disclosure. The anticipated consequences of HIV disclosure are recognised as important drivers in the disclosure process [2], while the fear of stigma is a known barrier stopping PLHIV from disclosing to people they are close to. The participants in the current study referred to the fear of stigma and the shame of a positive HIV diagnosis as their main reasons for delaying disclosure or not disclosing at all. Feelings of shame have been reported to inhibit HIV disclosure among older women living with HIV [49]. The older participants in this study had internalised the stigma and lived in shame and in fear that their HIV-positive status would be viewed even more shamefully because of their old age [49,50]. Several studies have identified the fear of stigma as a major factor that contributes to delayed disclosure or acts as the motivation for non-disclosure across population groups [18,19,20,34,51].

Secrecy and the selective disclosure are efforts to control the spread of gossip and are prominent manifestations of stigma among PLHIV [50]. Concerns about, and the fear of, rejection lead to the adoption and perpetuation of secrecy by PLHIV [2]. In the current study, the selection of a family member to disclose to was done in the hope that the person selected would keep the secret of the HIV diagnosis. In so doing, the participants not only sanctioned the secret nature of HIV diagnosis, but also made the family member to whom they had disclosed the keeper of the secret for a long period. Maman et al. [24] report similar observations.

Prior research conducted in South Africa has found that perceived stigma played a greater role in selective disclosure than the untoward negative reactions that the participants in those studies actually faced [7]. In the current study, some participants reported that their family members mistreated them after they disclosed. While they had initially delayed disclosure, when disclosure occurred it did so to persons they trusted, and from whom very little negative response was expected. However, they did receive actual stigma and discrimination. When they recounted their post-disclosure experiences, they narrated incidents of being discriminated against and rejected by their families. A recent longitudinal study in Zimbabwe found that disclosure to the family was associated with experiences of stigma [52].

As already stated, few (5/23) participants had disclosed to their sexual partners. Whereas disclosure to a partner is motivated by a desire to encourage the partner to get tested [24], none of their partners subsequently did so. Instead, disclosure led to negative consequences for their relationships. They experienced rejection from their partners, abandonment, being blamed for the infection, and stigma [5,24]. Consistent with prior studies, we found that the women participants did not receive support from their partners [24,49,52].

We found that most of the participants concealed their HIV status from their parents and non-family members. In addition, there was concealment of the HIV diagnosis from children. Only three of the participants, who were older women, had disclosed to their children, despite 18/28 of them having reported to be living with their children. Since the children were old enough to understand the meaning of a HIV-positive diagnosis [14], they disclosed to receive appropriate support in case their condition worsened. Nondisclosure is used by PLHIV as a mechanism to maintain privacy, which allows them to achieve some normality without the fear of rejection and stigma [43,50].

Of the 28 participants, five (three males and two females) had not disclosed their HIV-positive status to anyone, despite being hospitalised with AIDS-related opportunistic infections. They actively concealed their HIV-positive status because of their fear of stigma from their families and others. A review by Evangeli and Wroe [13] found that fear of rejection and discrimination from others is a global phenomenon for PLHIV. It is worrying that three of the participants who had not disclosed were in marital relationships. Beside the fact that concealment of one’s HIV status limits one’s opportunities to receive support [5], there are also implications for the transmission of HIV and reinfections if protective measures are not used. Non-disclosure can be a heavy psychological burden which can lead to feelings of anxiety and fear of social rejection [5]. In addition, non-disclosure can lead to feelings of isolation, depression, lack of social support, and poor medication adherence [13,53].

### Limitations

The findings of this study depended entirely on the participants’ reports. Hence, they cannot be generalised to the experiences of other people living with HIV in other settings.

## 5. Conclusions

Our findings corroborate previous studies by highlighting the relations that exist between perceived stigma, disclosure, and social support for PLHIV. The participants presented late for HIV diagnosis and medical care mainly due to their fear of receiving positive results and of HIV-related stigma. Consequently, all had been hospitalised with AIDS-related opportunistic illnesses. In addition, there is evidence of delayed and selective disclosure as well as widespread concealment of the HIV-positive status. We found that selective disclosure was deliberate and involved concealment of the HIV-positive status from family members to avoid negative emotional consequences following disclosure. There is a need for health care professionals to understand what informs disclosure or concealment of the HIV status of PLHIV for the effectiveness of interventions that seek to promote safe disclosure decisions.

The participants’ fear of stigma, rejection, and the shame of a HIV-positive diagnosis led to active concealment of their HIV serostatus from family, partners, and others. The implications of delayed disclosure, selective disclosure, and active concealment are the increased risk of the transmission of HIV, a lack of social support, and feelings of anxiety and fear of being socially rejected if one’s HIV status is revealed. The lack of social support might explain the poor levels of adherence prevalent among the participants in the study. It is therefore important that healthcare providers in primary health care settings take into consideration the interaction of HIV-related stigma and disclosure to develop disclosure-counselling strategies. These strategies should also address HIV-related stigma among family and community members.

The limitation of the counselling offered in HIV testing programs is that it is performed only once after an HIV positive test result, and does not take into considerations the short-term and long-term consequences of the diagnosis of HIV. The effect of counselling on the acceptance of a positive diagnosis is an area that has been neglected in research. Therefore, it is important to involve healthcare providers in research to create awareness of this limitation in the provision of HIV testing and counselling. This is crucial for the retention of people living with HIV in care.

## Figures and Tables

**Table 1:** Sociodemographic, clinical, and disclosure characteristics of the study sample.

Variable	Sub-Category	Freq.	Percentage
Gender	Female	18	64.3
	Male	10	35.7
Age category	20–39 years	13	46.4
	40–49 years	7	25.0
	50–60 years	8	28.6
Hospitalisation	First time	13	46.4
	Repeated admissions	15	53.6
Reason for testing	Pregnancy	3	10.7
	Partner died	1	3.6
	Partner was sick	4	14.2
	I was sick	20	71.4
Time since diagnosis with HIV	0–1 year	8	28.6
	2–5 years	7	25.0
	6–10 years	9	32.1
	More than 10 years	4	14.3
Duration on ART	0–1 year	13	46.4
	2–5 years	12	42.9
	6–10 years	3	10.7
Adherence to ART	Not adhering	10	35.7
	Adhering	18	64.3
Disclosed to one person	Yes	23	82.1
	No	5	17.9
First persons disclosed to	Close relative	10	43.5
	Parents	5	21.7
	Partner	5	21.7
	Children	3	13.1
Marital status	Widowed	2	7.1
	Married	11	39.3
	Single	15	53.6
Living arrangement	Parents and siblings	7	25
	Alone	3	10.7
	My partner and children	5	17.9
	My children only	11	39.3
Employment	Self-employed	3	10.7
	Employed	4	14.3
	Unemployed	21	75.0
Source of income	Old age pension	1	3.6
	No income	3	10.7
	Social grants (child and disability)	17	60.7
	Salary	7	25.0
Level of education	No formal education	2	7.1
	Primary education	4	14.3
	Secondary education	19	67.9
	Tertiary education	3	10.7

**Table 2:** Summary of themes and subthemes.

Theme	Subthemes
Delayed testing for HIV Selective disclosure of HIV status Endorsing the secret nature of the HIV diagnosis	
Delayed disclosure	Perceived stigma
	Self-stigma
	Disclosure delayed until symptoms were visible
	Disclosure delayed until after admission in hospital
Disclosure to receive appropriate care	
Negative responses to disclosure	

## Data Availability

All the data is included in the analysis of the study.
